# Type 1 Diabetes: A Disorder of the Exocrine and Endocrine Pancreas

**DOI:** 10.33696/immunology.5.177

**Published:** 2023

**Authors:** Brittany S. Bruggeman, Desmond A. Schatz

**Affiliations:** 1 University of Florida College of Medicine, Gainesville, Florida, USA

**Keywords:** Type 1 diabetes mellitus, Pancreas, Exocrine, Exocrine pancreatic insufficiency, Pancreatic elastase, Pancreatic alpha-amylases, Lipase, Trypsinogen

## Abstract

Type 1 diabetes has historically been described as an endocrine (β-cell) specific autoimmune disease. However, a substantial reduction (20–50%) in pancreas organ size and subclinical to symptomatic exocrine pancreatic insufficiency are present at diagnosis and may begin even prior to the development of islet autoimmunity. The mechanisms of exocrine loss in type 1 diabetes are not well understood, but leading hypotheses include developmental defects, β-cell loss resulting in exocrine atrophy, or autoimmune or inflammatory destruction of exocrine cells. Inflammatory changes including acute and chronic pancreatitis, exocrine T cell infiltration and classical complement activation, and serum exocrine autoantibodies within type 1 diabetes individuals suggest that an autoimmune or inflammatory process may contribute to exocrine pancreatic dysfunction. Exocrine pancreas atrophy primarily occurs prior to the onset of clinical disease. Indeed, recent work implicates exocrine-specific alterations in gene and protein expression as key in type 1 diabetes development. Measures of exocrine size and function could be useful additions in the prediction of disease onset and in identifying potential therapeutic responders to disease therapies, however, this is an underdeveloped area of research. Additionally, exocrine pancreatic insufficiency is underdiagnosed in individuals with type 1 diabetes and individualized treatment protocols are lacking. Much work remains to be done in this area, but we can definitively say that type 1 diabetes is a disorder of both the exocrine and endocrine pancreas likely from the start.

## Introduction

Type 1 diabetes (T1D) has traditionally been described as an endocrine (β-cell) specific autoimmune disease. However, significant exocrine pancreatic atrophy and fibrosis within the diabetic pancreas was first described one-hundred years ago [1]. Over sixty years ago reduced exocrine enzyme secretion and pancreas organ weight were found in patients with diabetes [2,3]. These findings were ascribed to loss of the trophic effects of insulin in the setting of β-cell loss and largely ignored for 50 years. Around 10 years ago the scientific community realized a renewed interest in T1D exocrine pancreatic pathology when Dr. Campbell-Thompson and colleagues found that islet autoantibody positive non-diabetic organ donors had reduced pancreas organ weight in addition to donors with established T1D [4]. This study was the first to demonstrate that exocrine dysfunction occurs early in the disease process, even prior to diagnosis of clinical disease, and may play a role in T1D pathogenesis.

Over the past 10 years our knowledge of exocrine pancreatic pathology in T1D has expanded. We now know that a substantial reduction (20–50%) in pancreas organ size and subclinical to symptomatic exocrine pancreatic insufficiency are present at diagnosis and may begin even prior to the development of islet autoimmunity [5–26]. Recent work implicates exocrine-specific alterations in gene and protein expression as key in T1D development [27,28]. While there is still much to learn, 100 years after the first description of exocrine pancreatic pathology we can definitively say that T1D is a disorder of both the exocrine and endocrine pancreas, likely from the start.

## Natural History of Exocrine Dysfunction in T1D

T1D progresses in stages: the development of islet autoimmunity (Stage 1 T1D) often occurs years prior to the loss of β-cell function (Stage 2 T1D), and clinical onset of disease (Stage 3 T1D) [29]. Exocrine pancreas atrophy appears to primarily occur prior to the onset of Stage 3 T1D, with some additional atrophy occurring through established disease, especially within the first year after diagnosis [30]. However, the course of events preceding clinical diagnosis of T1D is less clear [5–12,31]. There has been conflicting evidence showing reduced exocrine mass and function even prior to initial islet autoantibody seroconversion [5,6,30] versus between Stage 1 and 3 T1D [8,13,14]. The cross-sectional nature of most studies assessing exocrine changes in T1D is a major limitation.

The reduction in exocrine pancreatic function is largely subclinical, and changes in trajectory thus more meaningful [6,13,32]. To date, there has been only one longitudinal study of exocrine pancreatic function in the pre-clinical T1D period. Fecal elastase, the most used clinical marker of exocrine function, was prospectively evaluated in subjects at-risk for T1D within the Environmental Determinants of Islet Autoimmunity (ENDIA) birth cohort study [6]. Fecal elastase levels declined around the time of seroconversion in progressors to Stage 1–3 T1D but increased normally in non-progressors. Levels declined even prior to seroconversion in a substantial proportion of progressors. Baseline fecal elastase levels did not differ by progressor status or HLA (Human Leukocyte Antigen) type. The timing of exocrine dysfunction in pre-clinical T1D has potential implications for mechanism. For example, if function is reduced from birth in at-risk individuals, this may be due to a developmental defect caused by genetic factors or pathology in utero. If dysfunction occurs most prominently around the time of seroconversion, autoimmune or inflammatory mechanisms may be at play. And if occurring only after the onset of dysglycemia, exocrine atrophy may primarily occur due to deficiencies in exocrine-endocrine crosstalk [33]. Further longitudinal studies of exocrine pancreatic function and volume in at-risk subjects will clarify both the natural history and potential mechanisms for this phenomenon.

## Potential Underlying Mechanisms of Exocrine Dysfunction in T1D

The mechanisms of exocrine loss in T1D are not well understood, but leading hypotheses include developmental defects, insulinopenia, and more broadly endocrine deficiencies resulting in exocrine atrophy, or autoimmune or inflammatory destruction of exocrine cells [34,35].

### Deficiencies in endocrine-exocrine cross-talk as a contributor to exocrine atrophy

Reduced exocrine size and function in type 2 diabetes (T2D), though milder than in T1D, implicates insulinopenia as a potential mechanism. Some of the loss of exocrine pancreatic volume in T2D can be reversed with disease remission [36]. In T1D, pancreas volume continues to decline in the year post-diagnosis and very slowly thereafter [7,30]. Both findings further implicate insulinopenia as a contributor to exocrine atrophy. Supporting this notion is the finding that the body and tail of the pancreas in T1D, which have a greater proportion of β-cells, demonstrate greater atrophy than the head of the pancreas, with a greater proportion of pancreatic polypeptide (PP) cells [31].

Glucagon secretion is dysregulated in T1D, with an average increase in glucagon secretion but deficient release in hypoglycemic states. Chronic glucagon administration has generally been observed to lead to acinar atrophy and degranulation [37]. In one study, decreased α-cell mass was proportionately correlated to reductions in pancreatic weight in T1D organ donors [38]. Thus, dysregulated glucagon secretion in T1D may contribute to exocrine pancreatic atrophy, but up to this point this hypothesis has not been thoroughly investigated.

Recent data indicating a 57% reduction in acinar cell number, as opposed to cell size, in T1D donor pancreata suggest that insulinopenia and deficient endocrine-exocrine cross-talk does not entirely explain reduced exocrine mass in T1D [39]. Demonstrated exocrine abnormalities within first degree relatives of individuals with T1D and islet autoantibody positive subjects without dysglycemia also points towards a mechanism for exocrine changes outside of insulinopenia alone [5,6,30]. While congenital exocrine hypoplasia has been reported concomitantly with T1D in a case report, no other studies have demonstrated pancreatic developmental defects in individuals who go on to develop T1D [40]. However, a comparison of exocrine function or size at birth in healthy non-first degree-relative controls compared with individuals at-risk for T1D would better evaluate whether an inherent defect in pancreatic development could be causing any of the differences seen.

### Evidence for exocrine pancreatic autoimmunity

Previous studies have found autoantibodies against the exocrine pancreas to be present in 20–70% of subjects with T1D. These antibodies include those against carbonic anhydrase II (CAII), lactoferrin, pancreatic cytokeratin, bile salt-dependent lipase/carboxy ester lipase (BSDL/CEL), chymotrypsin, and amylase α−2A [41–46]. CAII and lactoferrin autoantibodies have been observed both in combination and as single autoantibodies in individuals with T1D, and in one study, were associated with reduced pancreatic function by BT-PABA excretion test [44]. These autoantibodies were not present in other non-pancreatic autoimmune processes, including ANA positivity, Graves’ disease, or rheumatoid arthritis [44–46]. Antibodies against BSDL/CEL and pancreatic cytokeratin are more common in T1D first degree relatives within the literature, but antibodies against CAII, lactoferrin, chymotrypsin, and amylase α−2A have not been previously evaluated within these populations [45,46]. Additionally, CD4 and CD8 T cell infiltrates and classical complement pathway activation are more common in the exocrine compartment than the islets in some studies of T1D pancreata [47,48].

**Figure 1.**
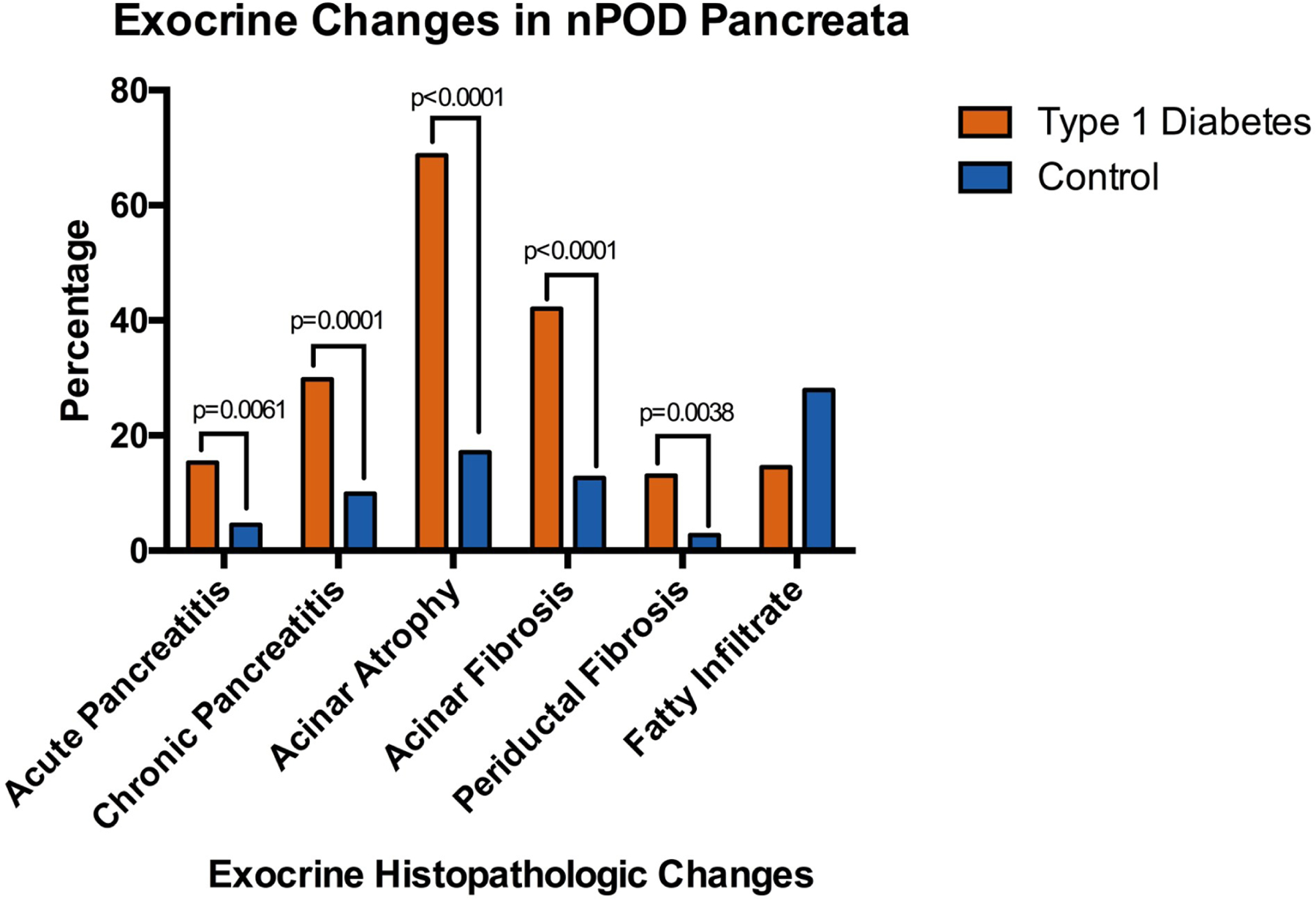
Percentage of type 1 diabetes (n=131) and control (n=111) pancreata from the Network for Pancreatic Organ donors with Diabetes (nPOD) who have histopathologic exocrine changes. Adapted from Bruggeman et al. [49].

We previously published an analysis of histopathologic exocrine changes within all Network for Pancreatic Organ donors with Diabetes (nPOD) T1D (n=131) and control (n=111) donor pancreata aged 12 and older available through November 2018. T1D donors were more likely to have acute (15.3% vs 4.5%, p=0.0061) and chronic pancreatitis (29.8% vs 9.9%, p=0.0001), acinar atrophy (68.7% vs 17.1%, p<0.0001), acinar fibrosis (42.0% vs 12.6%, p<0.0001), and periductal fibrosis (13.0% vs 2.7%, p=0.0038) (Figure 1), despite having comparable demographics including substance use patterns [49]. Taken together, these findings suggest that an autoimmune or inflammatory process may contribute to exocrine pancreatic loss and dysfunction present in T1D.

## Does Exocrine Dysfunction Play a Causal Role in T1D Pathogenesis?

A recent study of non-coding genetic risk variants in T1D exhibited enrichment in candidate *cis*-regulatory elements active in exocrine pancreatic acinar and ductal cells. In one example, a risk variant *cis*-regulatory element reduced ductal *cystic fibrosis transmembrane conductance regulator (CFTR)* expression. Another risk variant mapped to an acinar-specific region of chromatin directly upstream of the *CEL/BSDL* promoter; antibodies against this protein have been demonstrated in individuals both at risk for and with T1D [27,45]. Additionally, pathogenic genetic mutations in *CEL/BSDL* have been demonstrated in individuals with maturity-onset diabetes of the young type 8 (MODY8), neonatal-onset T1D, and atypical T2D [50]. Mendelian randomization studies of proteins influencing T1D susceptibility have implicated changes in exocrine protein expression, specifically chymotrypsinogen B1, as causal in T1D development [28]. While we have much to learn, recent studies with an agnostic approach to T1D pathogenesis have raised the supposition that dysregulated exocrine pancreatic gene and protein expression may be directly implicated in the development of disease rather than a simple byproduct of islet dysfunction.

## Using Exocrine Biomarkers in Disease Prediction & Monitoring

Large, well-designed birth cohort studies including the TEDDY (The Environmental Determinants of Diabetes in the Young) study have furthered our understanding of the events preceding β-cell autoimmunity (Stage 1 T1D), leading to loss of functional β-cell mass (Stage 2 T1D; dysglycemia), and clinical diabetes (Stage 3 T1D) [51]. However, heterogeneity in T1D onset as well as response to therapies calls for a better understanding of pathophysiology and additional markers of risk. Exocrine atrophy precedes the onset of detectable islet autoimmunity in some subjects [5,6,18,30], signifying that measures of exocrine pancreas size and function could be beneficial early biomarkers. If exocrine loss occurs before the onset of islet autoantibodies, markers of exocrine pancreas size or function could be useful to predict early T1D risk. If exocrine changes only occur in those imminently destined to develop clinical T1D, they could be useful to stratify risk in islet autoantibody positive subjects.

Serum trypsinogen and lipase levels can successfully categorize individuals as having ≥ 2 islet autoantibodies versus 1 and are an effective indicator of BMI-normalized relative pancreas volume by magnetic resonance imaging (MRI) [17]. Additionally, pancreas volume by MRI is smaller in T1D first degree relatives, islet autoantibody positive, and recent-onset T1D individuals versus controls respectively [5]. Exocrine serum, stool, or imaging markers could be integrated into existing T1D Risk Scores in combination with other already established biomarkers to further refine risk prediction [52,53]. Future studies should evaluate the utility of exocrine markers in prediction of disease onset and in identifying potential therapeutic responders to disease therapies.

## Diagnosis and Treatment of Exocrine Pancreatic Insufficiency in T1D

Exocrine pancreatic insufficiency (EPI) in T1D is common, present in approximately 33% of individuals versus 13% of the general population and is underdiagnosed. It is more common even than celiac disease or gastroparesis in the T1D population but is often forgotten when patients with T1D present with gastrointestinal complaints [54]. EPI clinically presents with symptoms of diarrhea or greasy stools, bloating, cramping, and/or weight loss. In individuals with T1D, it is associated with increased episodes of hypoglycemia [55]. Fecal elastase, an indirect test measuring levels of a proteolytic enzyme produced by acinar cells, is the most widely used clinically and is the most sensitive and specific non-invasive test available to detect EPI [6,13,20,56]. It is stable through intestinal passage, widely available, cost-effective, and requires only a single sample [57–61]. Importantly, it also correlates with reduced pancreatic volume in T1D [13,24] and has been validated for use in children [62,63]. Values <200 μg/g are supportive of EPI. Direct tests of pancreatic function (i.e., secretin-cholecystokinin test [64]), are the most accurate but are invasive, time-consuming, and expensive as they require sedation. Serum amylase, lipase, and trypsinogen are only sensitive for severe disease as reductions are typically subclinical and remain within the normal reference range [65].

Once diagnosed, EPI can be treated with Pancreatic Enzyme Replacement Therapy (PERT). However, much research remains to be done regarding the impact of PERT treatment on glycemic indicators and optimization of PERT dosing in the T1D patient population [54,66].

## Conclusions

While exocrine atrophy was first described over 100 years ago, in the past 10 years our understanding of T1D as a disorder of the exocrine and endocrine pancreas has evolved more than ever before. Signs of exocrine dysfunction arise early in the disease process, even prior to the development of clinical T1D. Early clues indicate that pathogenic alterations in exocrine gene regulation and protein expression may even play a causative role in T1D pathogenesis. Exocrine biomarkers hold promise for disease risk stratification and potentially delineation of responders to therapies. Clinically, individuals with T1D should be evaluated for EPI when they present with concerning symptoms and should be treated as needed with PERT. While our knowledge of this exciting and rediscovered facet of T1D has advanced, there is still much work to be done in all arenas of research spanning from bench to bedside; T1D researchers should consider this a call to action.

## Abbreviations:

BSDL/CEL: Bile Salt-dependent Lipase/Carboxy Ester Lipase
CAII: Carbonic Anhydrase II
EPI: Exocrine Pancreatic Insufficiency
MRI: Magnetic Resonance Imaging
PERT: Pancreatic Enzyme Replacement Therapy
T1D: Type 1 Diabetes
T2D: Type 2 Diabetes

## References

[1] Cecil RL. A STUDY OF THE PATHOLOGICAL ANATOMY OF THE PANCREAS IN NINETY CASES OF DIABETES MELLITUS. J Exp Med 1909 Mar 1;11(2):266–90. 19867248 10.1084/jem.11.2.266 PMC2124710

[2] Pollard HM, Miller L, Brewer WA. The external secretion of the pancreas and diabetes mellitus. Journ D. D 1943;10:20–3.

[3] MACLEAN N, OGILVIE RF. Observations on the pancreatic islet tissue of young diabetic subjects. Diabetes 1959 Mar-Apr;8(2):83–91. 13630179 10.2337/diab.8.2.83

[4] Campbell-Thompson M, Wasserfall C, Montgomery EL, Atkinson MA, Kaddis JS. Pancreas organ weight in individuals with disease-associated autoantibodies at risk for type 1 diabetes. JAMA 2012 Dec 12;308(22):2337–9. 10.1001/jama.2012.15008 23232891

[5] Campbell-Thompson ML, Filipp SL, Grajo JR, Nambam B, Beegle R, Middlebrooks EH, Relative Pancreas Volume Is Reduced in First-Degree Relatives of Patients With Type 1 Diabetes. Diabetes Care 2019;42(2):281–7. 30552130 10.2337/dc18-1512 PMC6341284

[6] Penno MAS, Oakey H, Augustine P, Taranto M, Barry SC, Colman PG, Changes in pancreatic exocrine function in young at-risk children followed to islet autoimmunity and type 1 diabetes in the ENDIA study. Pediatr Diabetes 2020 Sep;21(6):945–9. 32430977 10.1111/pedi.13056

[7] Virostko J, Williams J, Hilmes M, Bowman C, Wright JJ, Du L, Pancreas Volume Declines During the First Year After Diagnosis of Type 1 Diabetes and Exhibits Altered Diffusion at Disease Onset. Diabetes Care 2019;42(2):248–57. 30552135 10.2337/dc18-1507 PMC6341292

[8] Li X, Campbell-Thompson M, Wasserfall CH, McGrail K, Posgai A, Schultz AR, Serum Trypsinogen Levels in Type 1 Diabetes. Diabetes Care 2017;40(4):577–82. 28115475 10.2337/dc16-1774 PMC5360284

[9] Hardt PD, Krauss A, Bretz L, Porsch-Ozcürümez M, Schnell-Kretschmer H, Mäser E, Pancreatic exocrine function in patients with type 1 and type 2 diabetes mellitus. Acta Diabetol 2000;37(3):105–10. 11277309 10.1007/s005920070011

[10] Regnell SE, Peterson P, Trinh L, Broberg P, Leander P, Lernmark Å, Pancreas volume and fat fraction in children with Type 1 diabetes. Diabet Med 2016;33(10):1374–9. 26996278 10.1111/dme.13115

[11] Sasamori H, Fukui T, Hayashi T, Yamamoto T, Ohara M, Yamamoto S, Analysis of pancreatic volume in acute-onset, slowly-progressive and fulminant type 1 diabetes in a Japanese population. J Diabetes Investig 2018;9(5):1091–9. 10.1111/jdi.12816 PMC6123057 29427469

[12] Creutzfeldt W, Gleichmann D, Otto J, Stöckmann F, Maisonneuve P, Lankisch PG. Follow-up of exocrine pancreatic function in type-1 diabetes mellitus. Digestion 2005;72(2–3):71–5. 16113545 10.1159/000087660

[13] Augustine P, Gent R, Louise J, Taranto M, Penno M, Linke R, Pancreas size and exocrine function is decreased in young children with recent-onset Type 1 diabetes. Diabet Med 2020;37(8):1340–3. 31094026 10.1111/dme.13987

[14] Kondrashova A, Nurminen N, Lehtonen J, Hyöty M, Toppari J, Ilonen J, Exocrine pancreas function decreases during the progression of the beta-cell damaging process in young prediabetic children. Pediatr Diabetes 2018;19(3):398–402. 29044779 10.1111/pedi.12592

[15] Virostko J, Hilmes M, Eitel K, Moore DJ, Powers AC. Use of the Electronic Medical Record to Assess Pancreas Size in Type 1 Diabetes. PLoS One 2016;11(7):e0158825. 27391588 10.1371/journal.pone.0158825 PMC4938534

[16] Williams AJ, Thrower SL, Sequeiros IM, Ward A, Bickerton AS, Triay JM, Pancreatic volume is reduced in adult patients with recently diagnosed type 1 diabetes. J Clin Endocrinol Metab 2012;97(11):E2109–13. 22879632 10.1210/jc.2012-1815

[17] Ross JJ, Wasserfall CH, Bacher R, Perry DJ, McGrail K, Posgai AL, Exocrine Pancreatic Enzymes Are a Serological Biomarker for Type 1 Diabetes Staging and Pancreas Size. Diabetes 2021 Apr;70(4):944–54. 33441381 10.2337/db20-0995 PMC7980193

[18] Giovenzana A, Vecchio F, Cugnata F, Nonis A, Mandelli A, Stabilini A, Exocrine pancreas function is impaired in adult relatives of patients with type 1 diabetes. Acta Diabetol 2022 Apr;59(4):473–79. 34782929 10.1007/s00592-021-01819-2 PMC8917021

[19] Aughsteen AA, Abu-Umair MS, Mahmoud SA. Biochemical analysis of serum pancreatic amylase and lipase enzymes in patients with type 1 and type 2 diabetes mellitus. Saudi Med J 2005;26(1):73–7. 15756357

[20] Icks A, Haastert B, Giani G, Rathmann W. Low fecal elastase-1 in type I diabetes mellitus. Z Gastroenterol 2001;39(10):823–30. 11605150 10.1055/s-2001-17867

[21] Frier BM, Saunders JH, Wormsley KG, Bouchier IA. Exocrine pancreatic function in juvenile-onset diabetes mellitus. Gut 1976;17(9):685–91. 976808 10.1136/gut.17.9.685 PMC1411284

[22] Frier BM, Adrian TE, Saunders JH, Bloom SR. Serum trypsin concentration and pancreatic trypsin secretion in insulin-dependent diabetes mellitus. Clin Chim Acta 1980;105(2):297–300. 7398096 10.1016/0009-8981(80)90472-6

[23] Altobelli E, Blasetti A, Verrotti A, Di Giandomenico V, Bonomo L, Chiarelli F. Size of pancreas in children and adolescents with type I (insulin-dependent) diabetes. J Clin Ultrasound 1998;26(8):391–5. 9783245 10.1002/(sici)1097-0096(199810)26:8<391::aid-jcu3>3.0.co;2-d

[24] Philippe MF, Benabadji S, Barbot-Trystram L, Vadrot D, Boitard C, Larger E. Pancreatic volume and endocrine and exocrine functions in patients with diabetes. Pancreas 2011;40(3):359–63. 21283038 10.1097/MPA.0b013e3182072032

[25] Landin-Olsson M, Borgström A, Blom L, Sundkvist G, Lernmark A. Immunoreactive trypsin(ogen) in the sera of children with recent-onset insulin-dependent diabetes and matched controls. The Swedish Childhood Diabetes Group. Pancreas 1990;5(3):241–7. 2188253 10.1097/00006676-199005000-00001

[26] Ludvigsson J No acute pancreatitis but reduced exocrine pancreatic function at diagnosis of type 1 diabetes in children. Pediatr Diabetes 2019;20(7):915–9. 31392817 10.1111/pedi.12904

[27] Chiou J, Geusz RJ, Okino ML, Han JY, Miller M, Melton R, Interpreting type 1 diabetes risk with genetics and single-cell epigenomics. Nature 2021 Jun;594(7863):398–402. 34012112 10.1038/s41586-021-03552-w PMC10560508

[28] Yazdanpanah N, Yazdanpanah M, Wang Y, Forgetta V, Pollak M, Polychronakos C, Clinically Relevant Circulating Protein Biomarkers for Type 1 Diabetes: Evidence From a Two-Sample Mendelian Randomization Study. Diabetes Care 2022 Jan 1;45(1):169–77. 34758976 10.2337/dc21-1049

[29] Insel RA, Dunne JL, Atkinson MA, Chiang JL, Dabelea D, Gottlieb PA, Staging presymptomatic type 1 diabetes: a scientific statement of JDRF, the Endocrine Society, and the American Diabetes Association. Diabetes Care 2015;38(10):1964–74. 26404926 10.2337/dc15-1419 PMC5321245

[30] Wright JJ, Dulaney A, Williams JM, Hilmes MA, Du L, Kang H, Longitudinal MRI Shows Progressive Decline in Pancreas Size and Altered Pancreas Shape in Type 1 Diabetes. J Clin Endocrinol Metab 2023 Sep 18;108(10):2699–707. 36938587 10.1210/clinem/dgad150 PMC10505530

[31] Campbell-Thompson ML, Kaddis JS, Wasserfall C, Haller MJ, Pugliese A, Schatz DA, The influence of type 1 diabetes on pancreatic weight. Diabetologia 2016;59(1):217–21. 26358584 10.1007/s00125-015-3752-z PMC4670792

[32] Cavalot F, Bonomo K, Fiora E, Bacillo E, Salacone P, Chirio M, Does pancreatic elastase-1 in stools predict steatorrhea in type 1 diabetes? Diabetes Care 2006;29(3):719–21. 16505538 10.2337/diacare.29.03.06.dc05-1389

[33] Overton DL, Mastracci TL. Exocrine-Endocrine Crosstalk: The Influence of Pancreatic Cellular Communications on Organ Growth, Function and Disease. Front Endocrinol (Lausanne) 2022 Jun 13;13:904004. 35769082 10.3389/fendo.2022.904004 PMC9234176

[34] Foster TP, Bruggeman B, Campbell-Thompson M, Atkinson MA, Haller MJ, Schatz DA. Exocrine Pancreas Dysfunction in Type 1 Diabetes. Endocr Pract 2020 Dec;26(12):1505–13. 33471743 10.4158/EP-2020-0295 PMC8697709

[35] Campbell-Thompson M, Rodriguez-Calvo T, Battaglia M. Abnormalities of the Exocrine Pancreas in Type 1 Diabetes. Curr Diab Rep 2015;15(10):79. 26318606 10.1007/s11892-015-0653-y PMC5072278

[36] Al-Mrabeh A, Hollingsworth KG, Shaw JAM, McConnachie A, Sattar N, Lean MEJ, 2-year remission of type 2 diabetes and pancreas morphology: a post-hoc analysis of the DiRECT open-label, cluster-randomised trial. Lancet Diabetes Endocrinol 2020 Dec;8(12):939–48. 33031736 10.1016/S2213-8587(20)30303-X

[37] Alexandre-Heymann L, Mallone R, Boitard C, Scharfmann R, Larger E. Structure and function of the exocrine pancreas in patients with type 1 diabetes. Rev Endocr Metab Disord 2019 Jun;20(2):129–49. 31077020 10.1007/s11154-019-09501-3

[38] Bonnet-Serrano F, Diedisheim M, Mallone R, Larger E. Decreased α-cell mass and early structural alterations of the exocrine pancreas in patients with type 1 diabetes: An analysis based on the nPOD repository. PLoS One 2018 Jan 19;13(1):e0191528. 29352311 10.1371/journal.pone.0191528 PMC5774815

[39] Wright JJ, Saunders DC, Dai C, Poffenberger G, Cairns B, Serreze DV, Decreased pancreatic acinar cell number in type 1 diabetes. Diabetologia 2020;63(7):1418–23. 32388592 10.1007/s00125-020-05155-y PMC8403487

[40] Carroll PB, Finegold DN, Becker DJ, Locker JD, Drash AL. Hypoplasia of the pancreas in a patient with type I diabetes mellitus. Pancreas 1992;7(1):21–5. 1557342 10.1097/00006676-199201000-00004

[41] Endo T, Takizawa S, Tanaka S, Takahashi M, Fujii H, Kamisawa T, Amylase alpha-2A autoantibodies: novel marker of autoimmune pancreatitis and fulminant type 1 diabetes. Diabetes 2009;58(3):732–7. 19001184 10.2337/db08-0493 PMC2646073

[42] Hardt PD, Ewald N, Bröckling K, Tanaka S, Endo T, Kloer HU, Distinct autoantibodies against exocrine pancreatic antigens in European patients with type 1 diabetes mellitus and non-alcoholic chronic pancreatitis. JOP 2008;9(6):683–9. 18981548

[43] di Cesare E, Previti M, Lombardo F, Mazzù N, di Benedetto A, Cucinotta D. Prevalence of autoantibodies to carbonic anhydrase II and lactoferrin in patients with type 1 diabetes. Ann N Y Acad Sci 2004;1037:131–2. 15699506 10.1196/annals.1337.021

[44] Taniguchi T, Okazaki K, Okamoto M, Seko S, Tanaka J, Uchida K, High prevalence of autoantibodies against carbonic anhydrase II and lactoferrin in type 1 diabetes: concept of autoimmune exocrinopathy and endocrinopathy of the pancreas. Pancreas 2003;27(1):26–30. 12826902 10.1097/00006676-200307000-00004

[45] Panicot L, Mas E, Thivolet C, Lombardo D. Circulating antibodies against an exocrine pancreatic enzyme in type 1 diabetes. Diabetes 1999;48(12):2316–23. 10580419 10.2337/diabetes.48.12.2316

[46] Kobayashi T, Nakanishi K, Kajio H, Morinaga S, Sugimoto T, Murase T, Pancreatic cytokeratin: an antigen of pancreatic exocrine cell autoantibodies in type 1 (insulin-dependent) diabetes mellitus. Diabetologia 1990;33(6):363–70. 1696227 10.1007/BF00404641

[47] Rowe P, Wasserfall C, Croker B, Campbell-Thompson M, Pugliese A, Atkinson M, Increased complement activation in human type 1 diabetes pancreata. Diabetes Care 2013;36(11):3815–7. 24041678 10.2337/dc13-0203 PMC3816899

[48] Rodriguez-Calvo T, Ekwall O, Amirian N, Zapardiel-Gonzalo J, von Herrath MG. Increased immune cell infiltration of the exocrine pancreas: a possible contribution to the pathogenesis of type 1 diabetes. Diabetes 2014;63(11):3880–90. 24947367 10.2337/db14-0549 PMC4207385

[49] Bruggeman BS, Campbell-Thompson M, Filipp SL, Gurka MJ, Atkinson MA, Schatz DA, Substance Use Affects Type 1 Diabetes Pancreas Pathology: Implications for Future Studies. Front Endocrinol (Lausanne) 2021 Nov 29;12:778912. 34912300 10.3389/fendo.2021.778912 PMC8667172

[50] Wu H, Shu M, Liu C, Zhao W, Li Q, Song Y, Identification and characterization of novel carboxyl ester lipase gene variants in patients with different subtypes of diabetes. BMJ Open Diabetes Res Care 2023 Jan;11(1):e003127. 10.1136/bmjdrc-2022-003127 PMC9843195 36634979

[51] Rewers M, Hyöty H, Lernmark Å, Hagopian W, She JX, Schatz D, The Environmental Determinants of Diabetes in the Young (TEDDY) Study: 2018 Update. Curr Diab Rep 2018 Oct 23;18(12):136. 30353256 10.1007/s11892-018-1113-2 PMC6415767

[52] Orban T, Sosenko JM, Cuthbertson D, Krischer JP, Skyler JS, Jackson R, Pancreatic islet autoantibodies as predictors of type 1 diabetes in the Diabetes Prevention Trial-Type 1. Diabetes Care 2009;32(12):2269–74. 19741189 10.2337/dc09-0934 PMC2782989

[53] Sosenko JM, Skyler JS, Mahon J, Krischer JP, Beam CA, Boulware DC, The application of the diabetes prevention trial-type 1 risk score for identifying a preclinical state of type 1 diabetes. Diabetes Care 2012;35(7):1552–5. 22547092 10.2337/dc12-0011 PMC3379597

[54] Lewis DM. A Systematic Review of Exocrine Pancreatic Insufficiency Prevalence and Treatment in Type 1 and Type 2 Diabetes. Diabetes Technol Ther 2023 Sep;25(9):659–72. 37440180 10.1089/dia.2023.0157

[55] Basturk A, Curek Y, Felek R, Celmeli G, Artan R. Exocrine pancreas functions in children with type 1 diabetes mellitus. Arab J Gastroenterol 2021 Sep;22(3):236–9. 34509389 10.1016/j.ajg.2021.05.018

[56] Leeds JS, Oppong K, Sanders DS. The role of fecal elastase-1 in detecting exocrine pancreatic disease. Nat Rev Gastroenterol Hepatol 2011;8(7):405–15. 21629239 10.1038/nrgastro.2011.91

[57] Glasbrenner B, Schön A, Klatt S, Beckh K, Adler G. Clinical evaluation of the faecal elastase test in the diagnosis and staging of chronic pancreatitis. Eur J Gastroenterol Hepatol 1996;8(11):1117–20. 8944376 10.1097/00042737-199611000-00016

[58] Löser C, Möllgaard A, Fölsch UR. Faecal elastase 1: a novel, highly sensitive, and specific tubeless pancreatic function test. Gut 1996;39(4):580–6. 8944569 10.1136/gut.39.4.580 PMC1383273

[59] Stein J, Jung M, Sziegoleit A, Zeuzem S, Caspary WF, Lembcke B. Immunoreactive elastase I: clinical evaluation of a new noninvasive test of pancreatic function. Clin Chem 1996;42(2):222–6. 8595714

[60] Walkowiak J, Cichy WK, Herzig KH. Comparison of fecal elastase-1 determination with the secretin-cholecystokinin test in patients with cystic fibrosis. Scand J Gastroenterol 1999;34(2):202–7. 10192202 10.1080/00365529950173104

[61] Walkowiak J, Glapa A, Nowak JK, Bober L, Rohovyk N, Wenska-Chyży E, Pancreatic Elastase-1 Quick Test for rapid assessment of pancreatic status in cystic fibrosis patients. J Cyst Fibros 2016;15(5):664–8. 27287722 10.1016/j.jcf.2016.05.009

[62] Walkowiak J, Nousia-Arvanitakis S, Henker J, Stern M, Sinaasappel M, Dodge JA. Indirect pancreatic function tests in children. J Pediatr Gastroenterol Nutr 2005;40(2):107–14. 15699676 10.1097/00005176-200502000-00001

[63] Wieczorek-Filipiak M, Drzymała-Czyż S, Szczepanik M, Miśkiewicz-Chotnicka A, Wenska-Chyży E, Moczko JA, Fecal elastase-1 in healthy children up to 2 years of age: a cross-sectional study. Dev Period Med 2018;22(2):123–7. 30056398 10.34763/devperiodmed.20182202.123127 PMC8522897

[64] Law R, Lopez R, Costanzo A, Parsi MA, Stevens T. Endoscopic pancreatic function test using combined secretin and cholecystokinin stimulation for the evaluation of chronic pancreatitis. Gastrointest Endosc 2012;75(4):764–8. 22281107 10.1016/j.gie.2011.11.011 PMC4474136

[65] Lieb JG, Dragonov PV. Pancreatic function testing: here to stay for the 21st century. World J Gastroenterol 2008;14(20):3149–58. 18506918 10.3748/wjg.14.3149 PMC2712845

[66] Ewald N, Bretzel RG, Fantus IG, Hollenhorst M, Kloer HU, Hardt PD, Pancreatin therapy in patients with insulin-treated diabetes mellitus and exocrine pancreatic insufficiency according to low fecal elastase 1 concentrations. Results of a prospective multi-centre trial. Diabetes Metab Res Rev 2007 Jul;23(5):386–91. 17103488 10.1002/dmrr.708

